# Mouse monocytes express CD127 by immune cells, not LPS

**DOI:** 10.3389/fimmu.2024.1356004

**Published:** 2024-09-12

**Authors:** Reza Yazdani, Mozhde Askari, Amir Moghadam Ahmadi, Gholamreza Azizi, Bogoljub Ciric, Alexandra Boehm, Guang-Xian Zhang, Abdolmohamad Rostami

**Affiliations:** Department of Neurology, Thomas Jefferson University, Philadelphia, PA, United States

**Keywords:** IL-7 receptor, CD127, monocyte, macrophage, IL-7, inflammation

## Abstract

The essential role of interleukin 7 (IL-7) signaling via its receptor (IL-7Rα; CD127) in T cell development and function has been well documented. However, CD127 expression and function in myeloid cells, including monocytes, are less clear, especially in mice. In the present study we report an inducible CD127 expression in mouse monocytes/macrophages. This induction is dependent on the presence of other immune cells, highlighting that regulation of CD127 expression on monocytes differs in mice and humans. We demonstrate that CD127 is functional, as IL-7 downregulated its expression. We also saw decreased CD127 expression during inflammation *in vivo*. Overall, upregulation of CD127 expression *in vitro* and its downregulation *in vivo* confirm that CD127 is an inducible marker on mouse monocyte/macrophage cells, in contrast to findings recently published by others. Characterizing the role of CD127 signaling in myeloid cells in inflammatory disorders would be worthwhile in future study.

## 1 Introduction

Interleukin-7 (IL-7) is mainly produced by epithelial and stromal cells, and its function is mediated by the IL-7 receptor (IL-7R) (1). The IL-7R consists of two chains, the IL-7Rα chain (CD127) and common cytokine-receptor γ-chain (γc; CD132) (2). CD127 is expressed in lymphoid cell lineages, including T cells and innate lymphoid cells, in which the IL-7/IL-7R signaling promotes differentiation, survival, and homeostasis (3). Certain polymorphisms in the CD127 gene increase the risk of developing inflammatory disorders, including multiple sclerosis (MS), ulcerative colitis, and sarcoidosis (4), indicating that CD127 can contribute to the development of autoimmunity.

Most findings on the contribution of CD127 to various disorders are related to T cells, whereas only a few studies have investigated the role of CD127 in myeloid cells. Thus far, it has been reported that rheumatoid arthritis patients have a greater frequency of CD127+ macrophages than healthy subjects (5, 6), that lipopolysaccharide (LPS) treatment upregulates CD127 expression on human monocytes (7), and that increased CD127 expression on human monocytes is associated with some autoimmune diseases such as arthritis (8).

CD127 expression by human myeloid lineages has been evaluated, but studying its expression on mouse myeloid lineages is lagging. A recent study investigated the functional and molecular features of CD127-expressing monocytes in human and mouse system (9). The study found that human blood-derived monocytes expressed CD127 following LPS treatment, whereas mouse monocytes failed to do so. Given that CD127 could play a pathogenic role in inflammatory diseases such as MS (10, 11), we evaluated CD127 expression by mouse monocytes in steady state and in experimental autoimmune encephalomyelitis (EAE), an animal model of MS.

## 2 Materials and methods

### 2.1 Study design

We conducted this study on CD127 expression by mouse monocytes, and compared our results with a recent study conducted by Zhang et al. Furthermore, we functionally evaluated CD127 expression in response to the addition of IL-7 on CD127+ monocytes/macrophages. We also measured CD127 expression in naïve and mice with EAE to determine whether expression of this receptor changes in inflammatory conditions like EAE.

### 2.2 Mice

All mice used in this study were on C57BL/6J genetic background. Mice were obtained from The Jackson Laboratories (10–12 weeks old). C57BL/6J mice were housed in isolated, ventilated cages (maximum of 5 mice per cage) at the specific pathogen–free facility at Thomas Jefferson University. All experimental procedures were approved by the Institutional Animal Care and Use Committee of Thomas Jefferson University.

### 2.3 Human samples

Blood samples from healthy subjects were obtained at the Department of Neurology, Thomas Jefferson University. All subjects provided informed consent before their participation in the current study, and all human studies were approved by the Institutional Review Board (IRB) of Thomas Jefferson University. Consent forms were signed and reported yearly to Institutional Review Boards (IRB).

### 2.4 Cell isolation

Whole blood was collected into EDTA tubes (BD vacutainer system) and peripheral blood mononuclear cells (PBMCs) were obtained by density centrifugation (Ficoll Paque). The blood was first diluted 2-4 times with phosphate-buffered saline (PBS); the suspension was added to Ficoll-Paque solution at room temperature. After centrifugation at room temperature (1200 rpm, 30 min), the buffy coat layer containing the PBMC was collected, and a lysis step with ammonium chloride solution (Biolegend) was implemented to remove red blood cells (RBCs). For isolation of mononuclear cells from the spleen and lymph nodes of mice, tissues were homogenized by maceration through a 70 μm sterile filter with syringe plunger. Homogenized cells from the spleen were centrifuged at 1500 RPM (300 x g) for 10 min, followed by RBC lysis. To isolate CNS mononuclear cells, mice were perfused with ice-cold PBS, and brains and spinal cords were collected and digested in Liberase (Sigma-Aldrich) for 30 min at 37°C. Brains and spinal cords were then mechanically dissociated, and mononuclear cells were isolated using Percoll gradient (GE Healthcare).

### 2.5 Purification of monocytes

Monocytes were enriched by magnetic separation using an Isolation Kit (130-049-601, Miltenyi Biotec). Briefly, the PBMCs were magnetically labeled with CD11b Microbeads for mouse monocytes, and CD14 Microbeads for human monocytes. The cell suspension was then loaded onto a MACS^®^ Column which was placed in the magnetic field of a MACS Separator. The magnetically labeled cells were retained on the column. After removal of the column from the magnetic field, the magnetically retained labeled cells were eluted as the positively selected cell fraction. Isolated CD11b+ cells had a purity of almost 85% (**Supplementary Figure 1**).

### 2.6 Cell culture

Cells were rested overnight (16 hours) at 37°C, 5% CO_2_ in 5 ml non-adherent polypropylene cell-culture tubes (BD Biosciences) prior to stimulation assays. Given that we did not observe enough CD127+ mouse monocytes in plastic plates, probably due to monocyte adhesion to the plates, we used Falcon 352063 polypropylene tubes for this experiment. Cells were cultured in IMDM (Gibco) supplemented with 20% FBS (Gibco), and 1% penicillin/streptomycin with and without LPS (Escherichia coli O127:B8; Sigma-Aldrich) in a dosage of 100 ng/ml for mice and 10 ng/ml for human at 37°C under an atmosphere containing 5% CO_2_. After culture, the supernatants were removed, and monocytes were incubated in PBS containing 10 mM EDTA and 0.5% BSA on ice for 15 minutes to detach adherent cells. For the CD127 expression assay, we measured the receptor at 0, 6, and 18 hours. To assess CD127 expression following IL-7 addition, recombinant IL-7 (20 ng/ml; R&D Systems) was added for the last 2 hours of culture.

### 2.7 Induction and scoring of EAE

EAE was induced by immunization with 1:1 emulsion of PBS solution and complete Freund’s adjuvant (CFA) containing 5 mg/mL heat-killed *M. tuberculosis* (BD Biosciences) and 1 mg/mL MOG_35-55_ peptide (Genscript). Mice were immunized on both flanks by subcutaneous injection of the emulsion for a total of 200 µL. Pertussis toxin was intraperitoneally (i.p.) injected on days 0 and 2 post immunization (p.i.) at 200 ng per dose. Mice were scored according to the following scale: 0 - No clinical symptoms; 0.5 - Partial paralysis of the tail or waddling gait; 1.0 - Full paralysis of the tail; 1.5 - Full paralysis of the tail and waddling gait; 2.0 - Partial paralysis in one leg; 2.5 - Partial paralysis in both legs or one leg paralyzed; 3.0 - Both legs paralyzed; 3.5 - Ascending paralysis; 4.0 - Paralysis above the hips; 4.5 – Moribund; mouse being unable to right itself for 30 seconds; 5.0 - Death. EAE Mice were sacrificed at the peak of disease (14–15 days post-induction). Th score procedure was blindly done.

### 2.8 Antibodies

The fluorochrome-conjugated antibodies for targets of interest and fluorochrome-conjugated isotype control antibodies were as follows: anti-mouse CD3ϵ antibody [PE (clone 145-2C11, 100307; Biolegend), FITC (clone 145-2C11, 100306; Biolegend)], anti-mouse CD45 [Alexa Flour 700, clone I3/2.3, Biolegend], anti-mouse CD11b [Brilliant Violet 785 (clone M1/70, 101243; Biolegend), Brilliant Violet 421 (clone M1/70, 101251; Biolegend)], anti-mouse CD127 (IL-7Rα) [PE/Cyanine5 (clone A7R34, 135016; Biolegend), APC (clone A7R34, 135012,Biolegend), PE (clone A7R34, 135010; Biolegend)], anti-mouse Ly6C [Brilliant Violet 510 (clone HK1.4, 128033; Biolegend), Brilliant Violet 650 (clone HK1.4, 128049; Biolegend), Brilliant Violet 421 (clone HK1.4, 128032; Biolegend)], anti-mouse Ly6G [PE/Cyanine7, clone 1A8, 127618, Biolegend], anti-mouse I-A/I-E (MHCII) [PerCP/Cyanine 5.5 (clone M5/114.15.2, 107624; Biolegend), APC/Cyanine 7 (clone AF6-120.1, 116426; Biolegend)], anti-mouse F4/80 (MHCII) [PE/Cyanine 7, clone BM8, 123114; Biolegend], anti-mouse CD86 [FITC, clone GL-1, 105006, Biolegend], anti-mouse CD80 [PE, clone 16-10A1, 104708, Biolegend]. For human antibodies, the fluorochrome-conjugated antibodies were as follows: anti-human CD14 [APC/Cyanine 7, clone M5E2, 301820, Biolegend], anti-human CD14 [FITC, clone 3G8, 302006, Biolegend], anti-human CD3 [Brilliant Violet 650, clone OKT3, 317324, Biolegend], anti-mouse CD127 (IL-7Rα) [PE/Cyanine5 (clone A019D5, 351324; Biolegend). To optimize the concentration of antibodies used for flow cytometry experiments, titration of antibodies was performed.

### 2.9 Flow cytometry

Cells of interest were collected and washed with staining buffer (PBS containing 3% FBS). Cell surface antigens were stained with Abs in 100 μL of PBS/3%FBS for 20-30 min at 4°C. For blocking non-specific binding of immunoglobulin to the Fc receptors, we used FC blocker (TruStain FCx, Biolegend) before staining. After staining, cells were washed two times with staining buffer and resuspended in PBS/3%FBS. Cells were washed and fixed with 100 μL Fix and Perm Medium A (Thermo Fisher) for 20 min at room temperature and washed again. Cells were then washed twice and resuspended in 500 μL PBS, and data was obtained by FACSAria Fusion (BD Biosciences). Data analysis was implemented using FlowJo software (TreeStar).

### 2.10 Statistical analysis

Statistical comparison between two groups was done by student’s t-test and Mann-Whitney based on normality. One-way ANOVA was done for multiple group comparisons; ∗P < 0.05; ∗∗P < 0.01; ∗∗∗P < 0.001; NS, not statistically different. Prism 9.0 (GraphPad Software) was used for analyses.

## 3 Results

### 3.1 Mouse monocytes/macrophages express CD127

**Figure 1:**
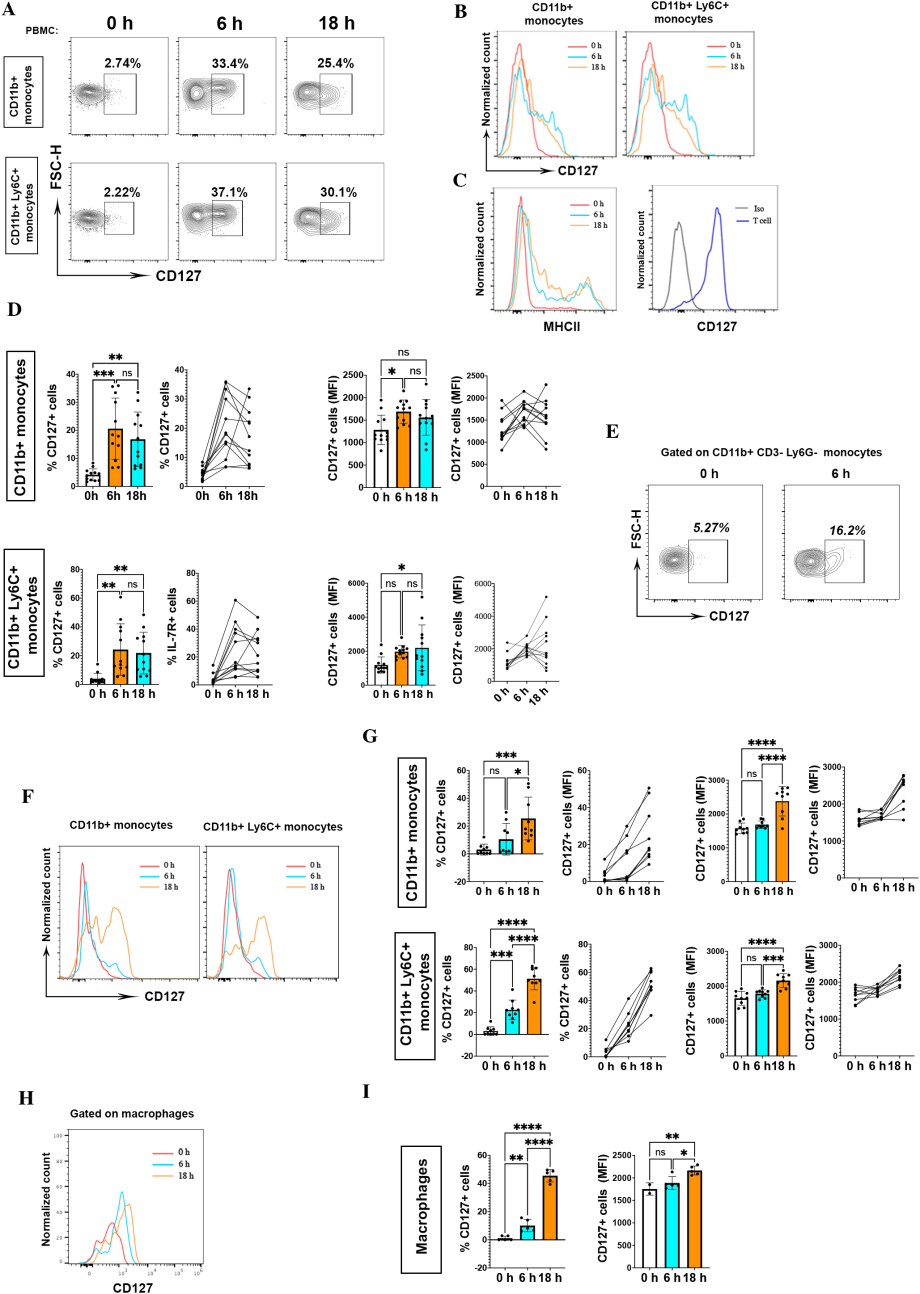
CD127 expression is upregulated on mouse monocytes/macrophages following LPS treatment. **(A)** CD127 expression was assessed on CD11b+ and CD11b+Ly6C+ mouse monocytes treated or not with 200 ng/ml LPS at different time points, as indicated. The top row shows CD127 expression on CD11b+ monocytes, and the bottom row on CD11b+ Ly6C+ monocytes. Cells were rested overnight (16 hours) at 37°C, 5% CO2 in 5 ml non-adherent polypropylene cell-culture tubes (BD Biosciences) prior to stimulation assays. **(B)** Histograms show CD127 expression at different time points. PBMCs from naïve mice were treated with LPS, then CD127 expression on untreated (0 h) and stimulated monocytes (6 h and 18 h) was measured. One representative result is shown. These experiments were conducted four times with similar outcomes (n= 12 mice). **(C)** To evaluate monocyte activation, MHCII expression on monocytes was measured at different time points as indicated. In the homeostatic condition, expression of CD127 in mouse T cells served as a positive control. One representative result is shown for MHCII expression and CD127 expression on T cell. **(D)** Statistical analysis of CD127 expression (percentage and MFI) between untreated (0 h) and stimulated (6 h and 18 h) CD11b+ and CD11b+ Ly6C+ monocytes. LPS 0 h, n= 12; LPS 6 h, n= 12; LPS 18 h, n= 12. *P < 0.05, **P < 0.01, and ***P < 0.001 by one-way ANOVA test (Tukey). NS, not significant. Graphs depict the mean ± SD. **(E)** CD127 expression on CD3- Ly6G- CD11b+ blood monocytes after 6 h LPS activation. One representative result is shown. **(F)** The same upregulation of CD127 expression after LPS activation (6 h and 18 h) in CD11b+ monocytes in the spleen. Left graph is related to CD127 expression on CD11b+ monocytes and right graph is for CD11b+ Ly6C+ monocytes at different time points. One representative result from one naïve mouse is shown. **(G)** CD127 expression in CD11b+ and CD11b+ Ly6C+ splenocytes was significant. These experiments were conducted two times with similar outcomes (n= 9 mice). *P < 0.05, **P < 0.01, ***P < 0.001, and ****P < 0.0001 by ANOVA test (Tukey). NS, not significant. Graphs depict the mean ± SD. **(H, I)** CD127 expression in macrophages (CD45+, CD11b+, Ly6C+, MHCII+, CD86+, CD80+) in different time points of stimulation (0h, 6h, 18h) (n= 5 mice). *P < 0.05, **P < 0.01, and ***P < 0.001 by one-way ANOVA test (Tukey). NS, not significant. Graphs depict the mean ± SD. *P < 0.05, **P < 0.01, ***P < 0.001, and ****P < 0.0001 by one-way ANOVA test (Tukey). NS, not significant. Graphs depict the mean ± SD.

We measured CD127 expression on mouse monocytes from blood at different time points during LPS stimulation (0, 6, 18 hours). Monocytes within LPS-stimulated PBMCs upregulated CD127 expression at 6 hours (20.5±10.9%) and 18 hours (16.8±9.7%) as compared to before stimulation [0 hours (4.15±2.1%)] (**Figures 1A B**). **Supplementary Figure 2** shows the detailed gating strategy for CD127 expression analysis on mouse monocytes. We measured CD127 expression on T cells as positive control, and assessed MHC II expression on monocytes, as an activation marker induced by LPS (**Figure 1C**). The upregulated CD127 on monocytes within LPS-stimulated PBMCs was significantly higher in terms of percentage and mean fluorescence intensity (**Figure 1D**). Zhang et al. reported that LPS induces CD127 expression on human monocytes, but not on murine monocytes (9). To better characterize murine monocytes, we also stained for Ly6C and found an inducible CD127 expression on blood CD11b+Ly6C+ monocytes after LPS activation (**Figures 1A, B**). Given that CD11b could be expressed on CD8+ T cells (12) and Ly6G+ neutrophils (13), we also determined CD127 expression on CD3-Ly6G-CD11b+ monocytes. Likewise, we found induced CD127 expression after LPS activation (**Figure 1E**).

CD127 expression on blood monocytes after LPS stimulation prompted us to evaluate its expression on splenic monocytes as well. Splenic monocytes also upregulated CD127 expression after LPS stimulation, especially after 18 hours of treatment (**Figures 1F, G**). Next, we analyzed CD127 expression on splenic macrophages (CD45+CD11b+Ly6C+MHCII+CD86+CD80+) at different time points (0, 6, 18 hours) after LPS treatment. Similarly, macrophages upregulated CD127 expression following LPS activation (**Figures 1H, I**).

Overall, we found that mouse blood and splenic monocytes can upregulate CD127 expression in culture containing LPS; however, if LPS is responsible for the CD127 upregulation needs to be addressed.

### 3.2 Mouse and human monocytes have different mechanisms involved in upregulating CD127 expression

**Figure 2:**
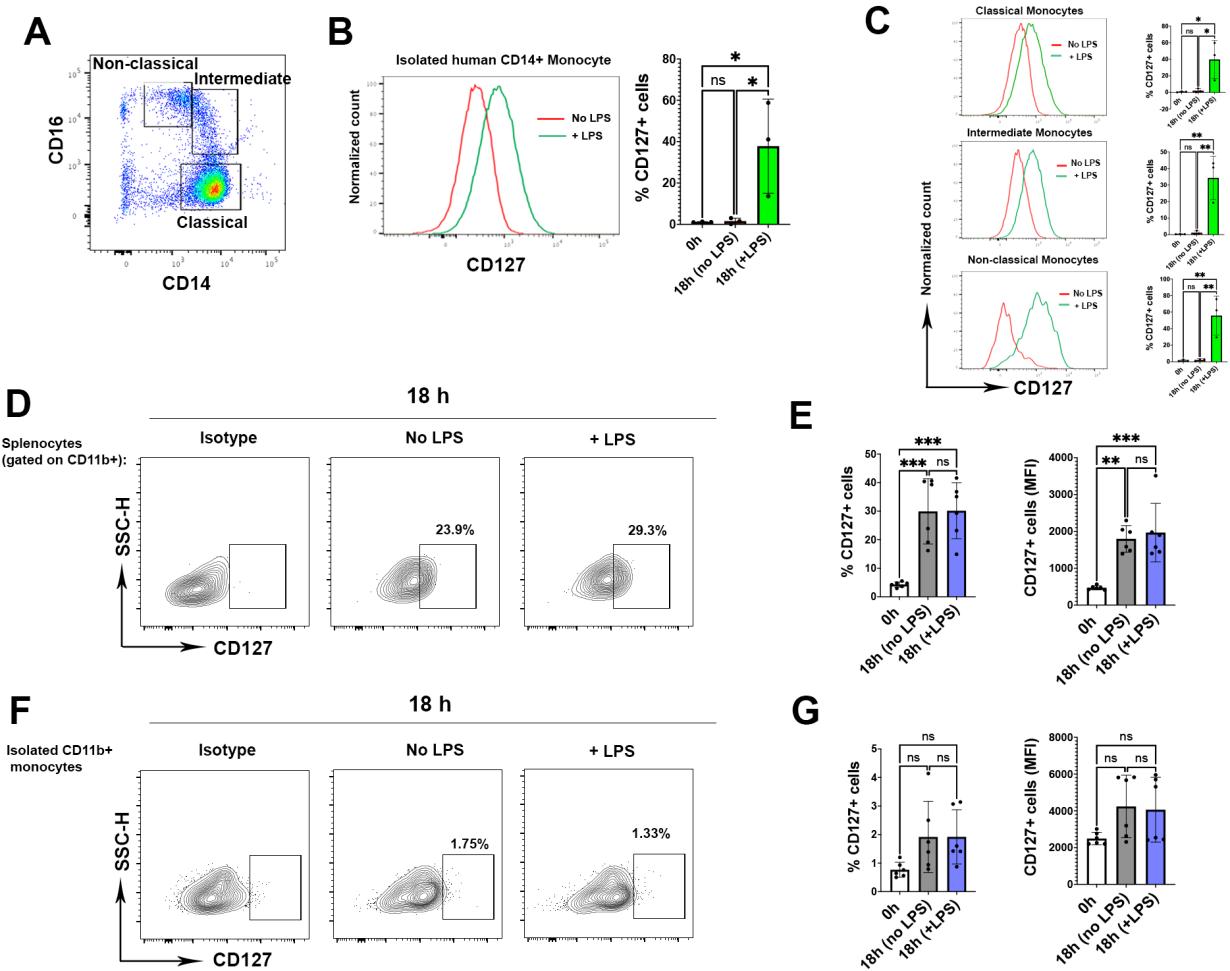
Monocytes upregulate CD127 expression in the presence of other immune cells. **(A)** The graph shows the gating strategy for classical (CD14+ CD16-), intermediate (CD14+CD16+) and non-classical (CD14-/low, CD16+) human monocytes. One representative FACS plot from three experiments is shown **(B)** CD127 expression on isolated human CD14+ monocytes following LPS activation. One representative FACS plot from three experiments is shown (n= 3 healthy controls, one-way ANOVA test) **(C)** CD127 expression in all types of human monocytes after LPS activation. The histograms are representative plots from three experiments (one-way ANOVA test) **(D)** The graphs gated on CD11b+ monocytes from one mouse where splenocytes were incubated for 18 h without (middle graph) or with LPS (right graph). Cells were rested overnight (16 hours) at 37°C, 5% CO2 in 5 ml non-adherent polypropylene cell-culture tubes (BD Biosciences) prior to stimulation assays. Left graph shows isotype controls for CD127 expression. One representative FACS plot from six experiments is shown. **(E)** Statistical analysis of the CD127 expression in both percentage (left graph) and mean fluorescence intensity (right graph) (n= 6 mice, one-way ANOVA test). **(F)** Isolated CD11b+ monocytes were cultured for 18 h without (middle graph) or with LPS (right graph). One representative FACS plot from six experiments is shown. **(G)** Statistical analysis of CD127 expression following culture compared to homeostatic condition (0 h) (n= 6 mice, one-way ANOVA test). *P* values were calculated using unpaired t test: *P < 0.05, **P < 0.01, and ***P < 0.001; NS, not significant. The analysis was conducted in two independent experiments (n = 6 mice). P values were calculated using by one-way ANOVA test (Tukey): *P < 0.05, **P < 0.01, and ***P < 0.001; NS, not significant.

Upregulation of CD127 expression on isolated human monocytes has been reported (8, 9). We also consistently observed upregulation of CD127 expression on isolated classic, intermediate and non-classic human monocytes after LPS activation (**Figures 2A–C**). Given that LPS induces CD127 expression on human monocytes, and we also found CD127 upregulation on mouse monocytes following LPS activation (**Figure 1**), we then tested if LPS is responsible for CD127 upregulation on mouse monocytes. Hence, we cultured splenic cells with or without LPS. Surprisingly, mouse monocytes upregulated CD127 expression in the splenic leukocyte cell mixture culture with and without LPS treatment compared with before culture (**Figures 2D, E**). CD127 expression was also upregulated in blood mouse monocytes that were not treated with LPS (**Supplementary Figure 3**). This finding is in contrast with the observations that human monocytes upregulated CD127 expression only after LPS treatment. We hypothesized that upregulation of CD127 on mouse monocytes may be induced by other splenic immune cells that present in the culture. To test this, we isolated CD11b+ monocytes and cultured them with or without adding LPS for 6 and 18 hours. Interestingly, we did not find any upregulation of CD127 expression irrespective of LPS stimulation (**Figures 2F, G**), indicating that upregulation of CD127 on mouse monocytes is induced by other immune cells. **Supplementary Figure 4** shows gating strategy for CD127 expression analysis on mouse CD11b+ and isolated CD11b+ cells from spleen.

### 3.3 Monocytes express functional CD127

**Figure 3:**
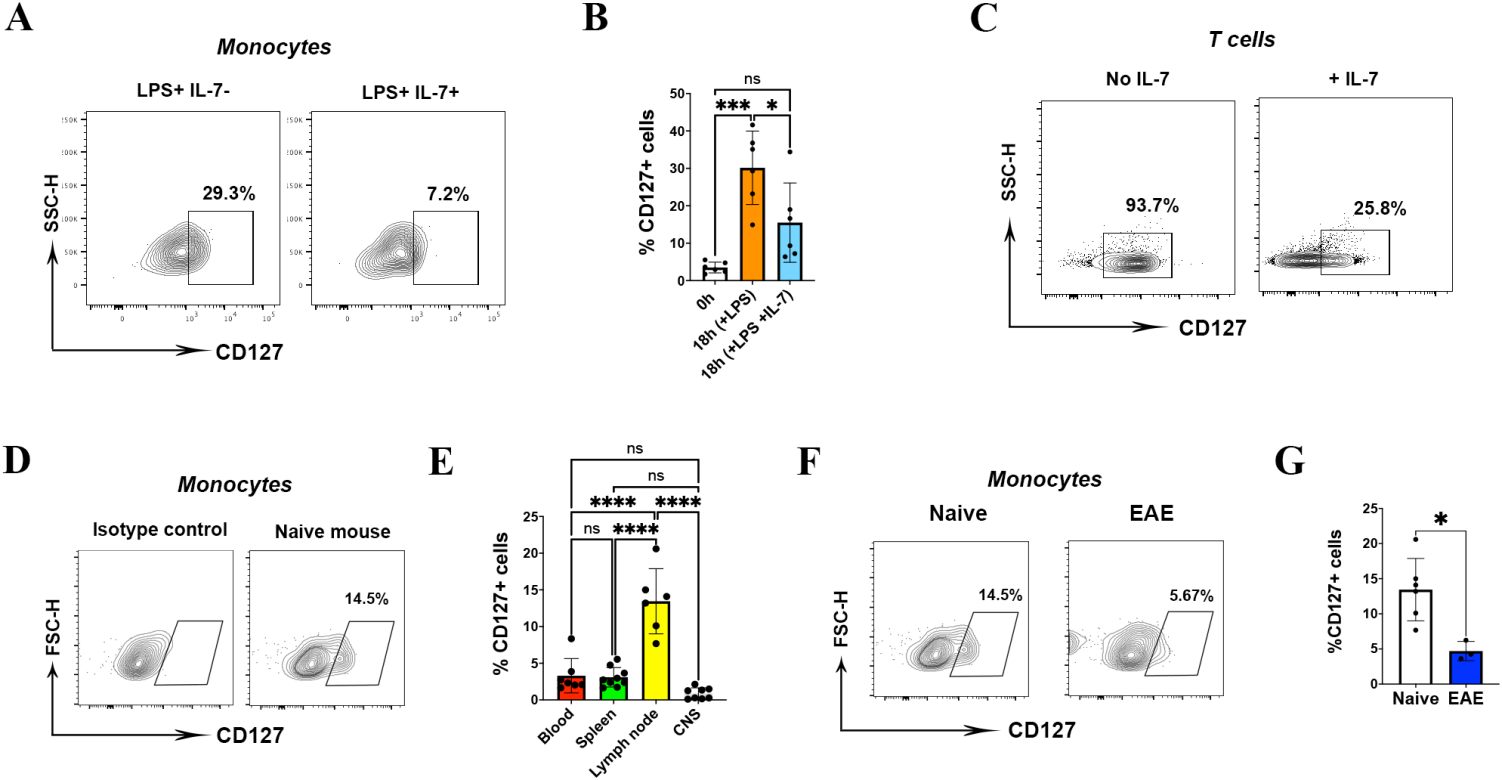
CD127 is downregulated on mouse monocytes during inflammation. **(A)** Plot demonstrating monocytes from splenocyte cultures either treated with LPS alone for 18 h, or with LPS with IL-7 added for the last 2 h of culture. One representative FACS plot from six experiments is shown. **(B)** Statistical analysis of CD127 expression following IL-7 treatment (n= 6 mice, one-way ANOVA test). **(C)** CD127 expression on IL-7-treated T cells served as a control. **(D)** The graph demonstrates CD127 expression on gated monocytes from lymph node of a naïve mouse at the homeostatic condition. **(E)** CD127 expression on different tissues (blood, spleen, lymph node and CNS) of naïve mice (n= 6 mice, one-way ANOVA test). **(F)** The graph demonstrates CD127 expression on gated monocytes from lymph nodes of a naïve mouse (left graph) and EAE mouse (right graph). **(G)** Statistical analysis of CD127 expression on EAE mice compared to naïve mice (n= 5 mice, unpaired t-test). *P* values were calculated using unpaired t test: *P < 0.05, ***P < 0.001, and ****P < 0.0001; NS, not significant. All experiments in naïve and EAE were conducted two times.

To examine whether upregulated CD127 expression on mouse monocytes are functional, we treated them with recombinant IL-7 (20 ng/ml). IL-7 treatment caused a significant loss of CD127 from the surface of monocytes (**Figures 3A, B**). As a control, we treated T cells with IL-7 and observed similar reduction in CD127 levels (**Figure 3C**).

### 3.4 Downregulation of CD127 expression on mouse monocytes following IL-7 treatment and during inflammation

We next explored whether CD127 expression on mouse monocytes changes during inflammation *in vivo*. Given that CD127 is expressed at the minimal level on blood and splenic monocytes in homeostatic conditions, we assessed CD127 expression on CNS and lymph node monocytes as well. We have not found CD127 expression on CD11b+ cells from the CNS, including microglia; surprisingly, we have detected CD127 on lymph node monocytes of naïve mice (**Figure 3D**). CD127 was expressed significantly higher on lymph node monocytes (mean=13.4%) than on those from the blood (mean=3.2%), spleen (mean=3.0%) and CNS (mean=0.8%) in steady state (**Figure 3E**). Hence, we compared CD127 expression of lymph node monocytes between naïve and mice with EAE. We immunized naïve mice with MOG_35-55_ peptide and *M. tuberculosis* to induce EAE, and collected cells at the peak of disease. Compared with naïve mice, the CD127 expression was significantly lower in lymph node monocytes of mice with EAE (**Figures 3F, G**). There was no significant change of CD127 expression on spleen, blood and CNS monocytes of mice with EAE (data not shown).

## 4 Discussion

In this study, we found that different mechanisms involved in upregulating CD127 expression in mouse and human monocytes, as LPS is not an activator for CD127 expression in mice, in contrast to human monocytes. Our data showed that the pattern of CD127 expression is different in mouse monocytes in different tissues, as only lymph nodes express CD127 at homeostatic condition. Downregulation of CD127 expression on inflammatory condition highlights a role of this receptor in health and disease.

Reports on CD127 expression by mouse myeloid cells are rare. Leung et al. reported an upregulation of *Il7ra* mRNA expression and CD127 surface protein levels upon transition of monocytes to macrophages within fetal tissues in mice (14). Another study showed that CD127 expression was upregulated on human and mouse macrophages following LPS treatment *in vitro*, and that macrophages also upregulated CD127 after LPS was intraarticularly injected in mice (15). A recent study indicated CD127 expression was upregulated by CHBP (an inhibitor of macrophage inflammation and DCs maturation) on mouse CD11b+Ly6G-Ly6C+ myeloid-derived suppressor cells (MDSCs), and CD127+ monocytic MDSCs had stronger immunosuppressive capacity than CD127− monocytic MDSCs (16). These findings show that mouse monocytes/macrophages could express CD127 after activation. To the best of our knowledge, no report other than one by Zhang et al. about CD127 expression analysis on mouse monocytes *in vitro* after activation exists. Upregulation of CD127 expression has been shown on human monocytes after LPS activation (8), highlighting that both human and mouse monocytes can express CD127.

Cell surface markers present on cells *in vivo* may not be present on monocytes maintained under various culturing conditions *in vitro* (17). We therefore used polypropylene cell-culture tubes for *in vitro* culture. We found inducible CD127 expression on monocytes when we cultured them in polypropylene tubes. Different result between our study and that of Zhang et al. could be attributed to differences in culturing conditions (9). Increased CD127 expression after LPS activation demonstrates that CD127 is an inducible marker in monocytic cells, in contrast to T cells that constitutively expresses this receptor (18, 19). Together these results demonstrate that CD127 expression can be induced in mouse and human monocytes. Our data on mouse monocytes, together with reports on human monocytes, highlight that they both can be induced to express CD127. However, mechanisms involved in upregulation of CD127 expression in mouse and human monocytes are different. Upregulation of CD127 expression on mouse monocytes without LPS stimulation shows that LPS is not an activator for CD127 expression in mice, in contrast to human monocytes. Increased CD127 expression on mouse monocytes in a cell mixture could be caused by cell-cell interaction or cytokine production by other immune cells.

Studies have demonstrated that mouse and human T cells transiently downregulate CD127 expression after encountering IL-7 (20). There are no reports on the impact of IL-7 on CD127 expression on mouse monocytes. Regarding CD127 downregulation on T cells, it is assumed that constitutes a homeostatic mechanism that regulates IL-7 consumption by naive T cells for survival (20, 21). Consistent with our result on mouse monocytes, one study has reported that IL-7 reduces CD127 expression on human monocytes (8). Our finding that IL-7 treatment reduces CD127 levels on mouse monocytes indicates that CD127 could be functional.

Decreased CD127 expression on lymph node monocytes in EAE could be related to the immunomodulatory role of CD127 during inflammation, as it has been reported that the presence of CD127+ monocytes/macrophages probably correlates with subdued inflammation (9). Consistent with our data, it has been reported that the expression level of CD127 in peripheral blood of MS patients is lower than in healthy controls (22). This reduction was also observed in T cells of MS patients compared to healthy individuals (23). However, there are no reports on CD127 expression in monocytes of animals with EAE and MS patients. Hence, we believe that characterizing the role of CD127 on monocytes in EAE and in MS patients would advance our understanding of disease pathogenesis.

## 5 Conclusions

Information about the expression of CD127 in mouse monocytes/macrophages is sparse. In this study, we demonstrated that CD127 expression in mouse monocytes is inducible, like human monocytes. However, regulation of CD127 expression on mouse and human monocytes differs, as CD127 expression on human monocyte is induced by LPS treatment, whereas in mouse monocytes is not. Furthermore, we demonstrated that CD127 on mouse monocytes could be functional, as IL-7/CD127 interaction downregulated CD127 expression. Certain polymorphisms in the CD127 gene increase risk for MS patients, however, its expression and function on monocytes are unclear. Characterizing the role of CD127 signaling in EAE and MS would help to determine if CD127 is a promising target for MS therapy. Further studies on transgenic mice with altered CD127 gene expression/activity will provide answers regarding the role of CD127+ monocytes in inflammatory disorders such as MS.

## Data Availability

The raw data supporting the conclusions of this article will be made available by the authors, without undue reservation.

## Supplementary material

Supplementary Figure 1 The purity of splenic CD11b+ cells isolated with MACS. Monocytes were first gated based on cell size (forward scatter) and internal granularity (side scatter) **(A)**. After excluding unwanted events and doublets **(B, C)**, live cells were gated to analyze purity of CD11b+ cells **(D)**. The percentage of CD11b+ cells is shown by histogram plot **(E)**.

Supplementary Figure 2 Gating strategy for CD127 expression on mouse monocytes following LPS treatment. Monocytes were first gated based on cell size (forward scatter) and internal granularity (side scatter) **(A)**. After excluding unwanted events and doublets **(B)**, monocytes were gated among CD45high and CD11b+ cells **(C)**. The percentage of CD127+ cells among CD11b+ cells **(D)** and CD11b+Ly6C+ **(E, F)** cells was analyzed. Top row is related to CD11b+ monocytes gating strategy and lower row is related to gating strategy gate of CD11b+Ly6C+ monocytes. The same gating strategy was applied for 6 h and 18 h in **Figure 1E**.

Supplementary Figure 3 Gating strategy for CD127 expression on mouse blood monocytes without LPS treatment. Monocytes were first gated based on cell size (forward scatter) and internal complexity (side scatter) **(A)**. After excluding unwanted events and doublets **(B)**, monocytes were gated among CD45high and CD11b+ cells **(C)**. The percentage of CD127+ cells in CD11b+ cells was analyzed at 0 h (top), 6 h without LPS (middle) and 6 h with LPS (below).

Supplementary Figure 4 Gating strategy for CD127 expression on mouse CD11b+ splenocytes and isolated CD11b+ splenic cells. Monocytes were gated based on cell size (forward scatter) and internal granularity (side scatter) **(A)**. After excluding unwanted events and doublets **(B, C)**, alive cells were gated for analysis. Monocytes were gated among CD3- and CD11b+ cells **(C)**. The percentage of CD127+ cells in CD11b+ (top) and isolated CD11b+ (below) splenocytes was analyzed. Top and below rows are related to 18 h culture without LPS. The same gating strategy was applied for analyses of 18 h culture treated with LPS on mouse CD11b+ and isolated CD11b+ splenocytes.
