# Supplementary figures and images for: Mouse monocytes express CD127 by immune cells, not LPS
Source: Front Immunol. 2024 Sep 12;15:1356004. doi: 10.3389/fimmu.2024.1356004 (PMC11424446; doi:10.3389/fimmu.2024.1356004)

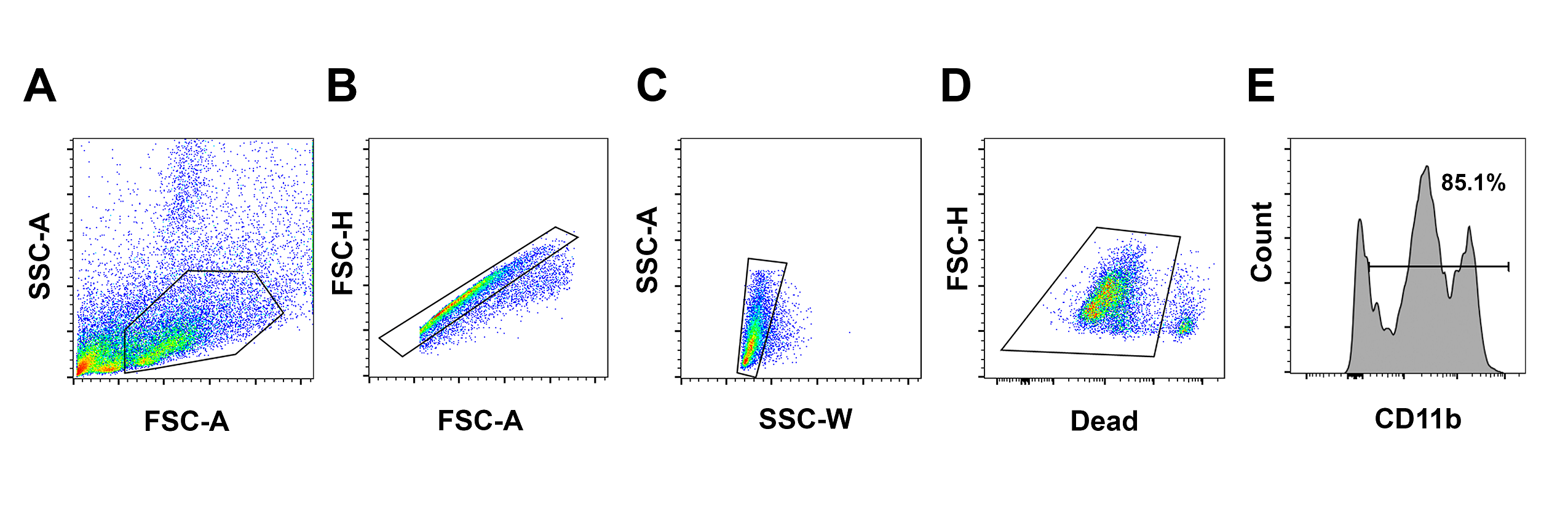

Supplement: Supplementary Figure 1 — The purity of splenic CD11b+ cells isolated with MACS. Monocytes were first gated based on cell size (forward scatter) and internal granularity (side scatter) (A). After excluding unwanted events and doublets (B, C), live cells were gated to analyze purity of CD11b+ cells (D). The percentage of CD11b+ cells is shown by histogram plot (E). [file Image1.tif]

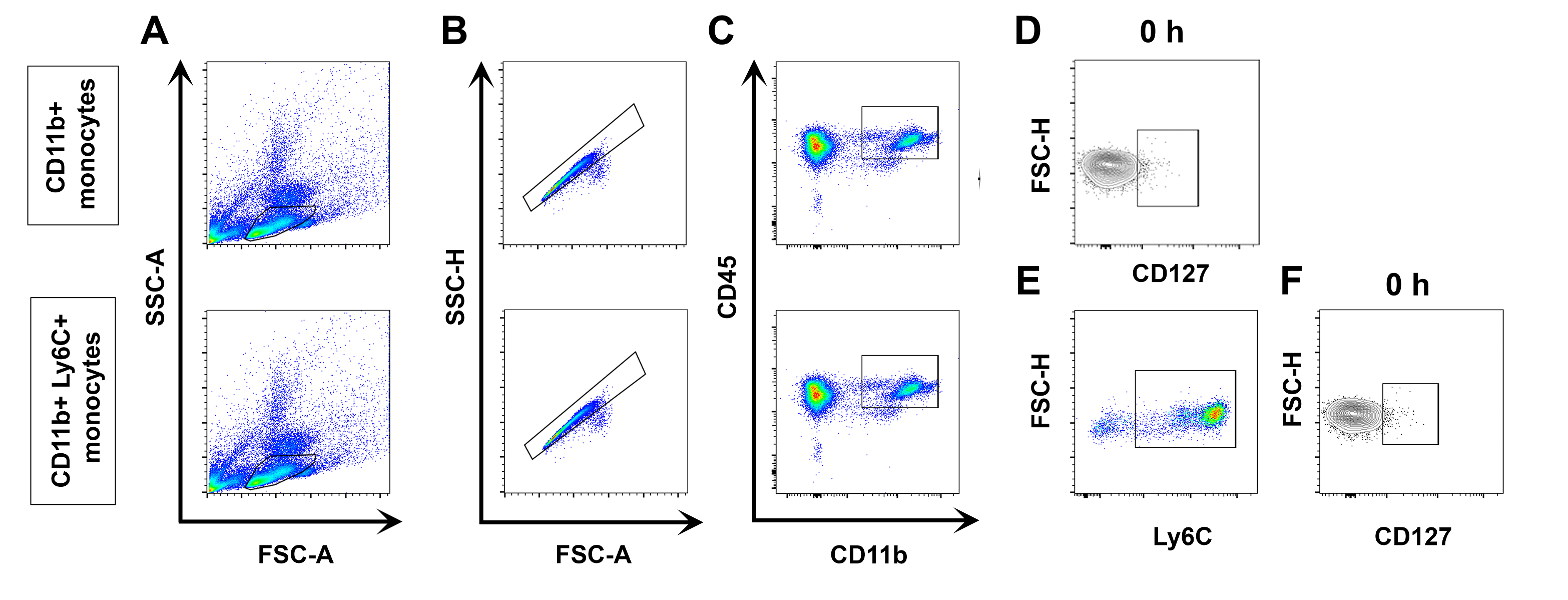

Supplement: Supplementary Figure 2 — Gating strategy for CD127 expression on mouse monocytes following LPS treatment. Monocytes were first gated based on cell size (forward scatter) and internal granularity (side scatter) (A). After excluding unwanted events and doublets (B), monocytes were gated among CD45high and CD11b+ cells (C). The percentage of CD127+ cells among CD11b+ cells (D) and CD11b+Ly6C+ (E, F) cells was analyzed. Top row is related to CD11b+ monocytes gating strategy and lower row is related to gating strategy gate of CD11b+Ly6C+ monocytes. The same gating strategy was applied for 6 h and 18 h in Figure 1E . [file Image2.tif]

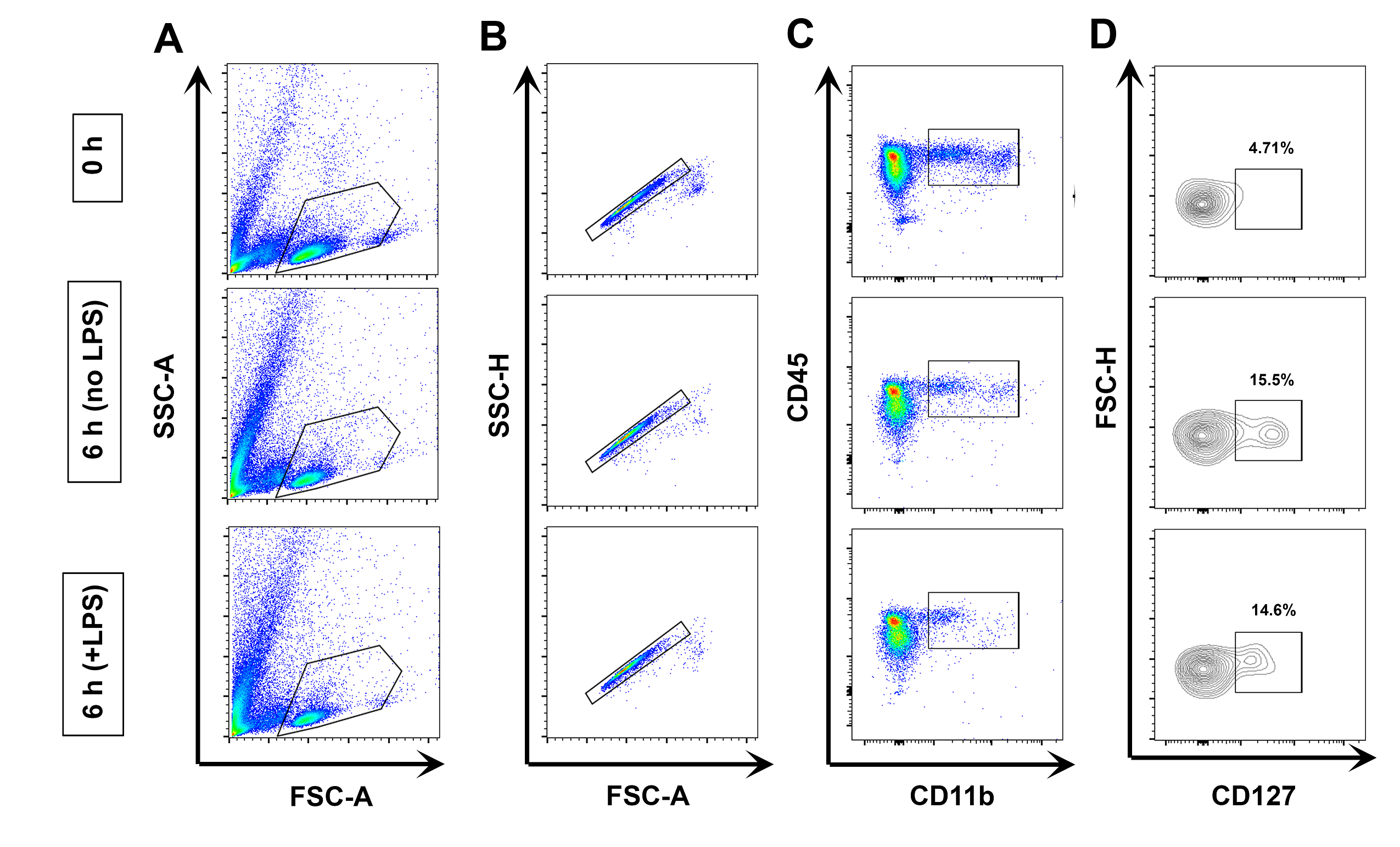

Supplement: Supplementary Figure 3 — Gating strategy for CD127 expression on mouse blood monocytes without LPS treatment. Monocytes were first gated based on cell size (forward scatter) and internal complexity (side scatter) (A). After excluding unwanted events and doublets (B), monocytes were gated among CD45high and CD11b+ cells (C). The percentage of CD127+ cells in CD11b+ cells was analyzed at 0 h (top), 6 h without LPS (middle) and 6 h with LPS (below). [file Image3.tif]

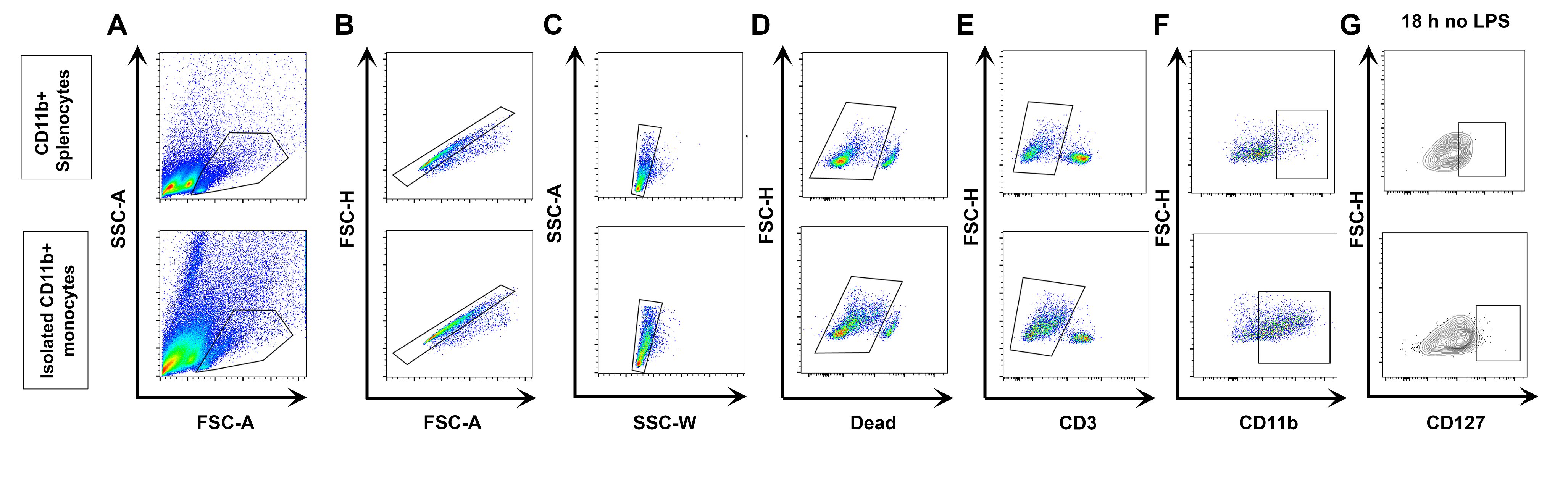

Supplement: Supplementary Figure 4 — Gating strategy for CD127 expression on mouse CD11b+ splenocytes and isolated CD11b+ splenic cells. Monocytes were gated based on cell size (forward scatter) and internal granularity (side scatter) (A). After excluding unwanted events and doublets (B, C), alive cells were gated for analysis. Monocytes were gated among CD3- and CD11b+ cells (C). The percentage of CD127+ cells in CD11b+ (top) and isolated CD11b+ (below) splenocytes was analyzed. Top and below rows are related to 18 h culture without LPS. The same gating strategy was applied for analyses of 18 h culture treated with LPS on mouse CD11b+ and isolated CD11b+ splenocytes. [file Image4.tif]
